# Exploring the Link Between Nutritional and Functional Status and Short-Term Postoperative Outcomes in Patients Undergoing Pancreatic Cancer Surgery

**DOI:** 10.3390/life15050803

**Published:** 2025-05-18

**Authors:** Irene Lidoriki, Maximos Frountzas, Eva Karanikki, Adam Mylonakis, Alexandros Kozadinos, Iliana Tsikrikou, Maria Kyriakidou, Lysandros Karydakis, Dimitrios Stefanoudakis, Maria Lampou, Michail Vailas, Evangelos Felekouras, Konstantinos G. Toutouzas, Dimitrios Schizas

**Affiliations:** 1 Department of Environmental Health, Harvard T.H. Chan School of Public Health, Boston, MA 02115, USA; 2 Department of Occupational Medicine, Cambridge Health Alliance, Cambridge, MA 02145, USA; 3 First Propaedeutic Department of Surgery, National and Kapodistrian University of Athens, Hippocration General Hospital, 11527 Athens, Greece; froumax@med.uoa.gr (M.F.); karanikki.eva@gmail.com (E.K.); dimitristef99@gmail.com (D.S.); mike_vailas@yahoo.com (M.V.); tousur@med.uoa.gr (K.G.T.); 4 First Department of Surgery, National and Kapodistrian University of Athens, Laikon General Hospital, 11527 Athens, Greece; adam.mylonakis@med.uoa.gr (A.M.); akozadinos@med.uoa.gr (A.K.); il.tsikrikou@gmail.com (I.T.); maria.ath.2007@gmail.com (M.K.); lys_karydakis@yahoo.gr (L.K.); felek@med.uoa.gr (E.F.); dschizas@med.uoa.gr (D.S.); 5 Division of Infectious Diseases, Massachusetts General Hospital, Harvard Medical School, Boston, MA 02114, USA

**Keywords:** nutritional status, functional status, pancreatic cancer, complications, morbidity, mortality, length of hospital stay

## Abstract

Background: Patients with pancreatic cancer are at increased risk of malnutrition and functional decline. This study aimed to identify preoperative nutritional and functional risk factors associated with postoperative complications. Methods: Adult patients with pancreatic cancer admitted to two university surgical departments in Athens, Greece, were included. Preoperative laboratory values, nutritional status, and functional status were assessed. Results: Among 96 patients included in this study, postoperative complications occurred in 69 (71.9%). Complication rates were significantly higher in patients with severe malnutrition (87.9% vs. 62.7%, *p* = 0.015), low Geriatric Nutritional Risk Index (GNRI) (90.5% vs. 66.2%, *p* = 0.023), low handgrip strength (91.7% vs. 61.5%, *p* = 0.037), and low gait speed (90.5% vs. 63.6%, *p* = 0.027). Major complications were linked to lower hemoglobin (Hb) (11.5 ± 1.9 vs. 12.8 ± 1.7 g/dL, *p* = 0.001), lower hematocrit (HCT) (34.7 ± 5.4% vs. 38.4 ± 4.7%, *p* = 0.001), and severe malnutrition (42.4% vs. 23.7%, *p* = 0.05). Lower Hb, lower HCT, malnutrition, and greater waist circumference were associated with postoperative mortality. Longer hospital stays were correlated with weaker handgrip strength and slower gait speed. Conclusion: Preoperative malnutrition and impaired functional status are significant predictors of adverse postoperative outcomes in pancreatic cancer surgery.

## 1. Introduction

Pancreatic tumors can be divided into two large categories, exocrine pancreatic cancer, which includes adenocarcinoma, and pancreatic neuroendocrine tumors. Pancreatic ductal adenocarcinoma accounts for more than 90% of pancreatic cancers. The incidence of pancreatic cancer is not high, whereas survival rates are low, with pancreatic cancer having one of the highest mortality rates and an overall survival rate of 6% [1]. In general, pancreatic cancer patients with localized disease present an approximately 40% chance of 5-year survival, whereas those with metastatic disease only 2–3% [2,3].

Surgery is the treatment of choice for patients suffering from pancreatic cancer. Surgical procedures may include pancreatoduodenectomy, distal or total pancreatectomy. Such complex operations are usually accompanied by a great risk of postoperative complications that, in turn, might be associated with poor patient prognosis [4]. However, contemporary pancreatic cancer management includes a multimodal therapeutic approach, which often combines perioperative chemotherapy and/or radiotherapy.

Malnutrition is a common finding in patients with pancreatic cancer, increasing the risk of adverse postoperative outcomes [5,6]. A significant percentage of patients report loss of appetite, abdominal pain, vomiting, dysphagia, malabsorption, and changes in bowel movements, which lead to inadequate nutritional intake, weakness, and marked functional decline. As a result, pancreatic cancer patients, especially those who undergo major surgery combined with perioperative chemotherapy, experience unintentional weight loss and decreased muscle mass [7,8]. Sarcopenia and cachexia are also highly prevalent in cancer patients, rendering worse quality of life, increased vulnerability, a decrease in physical strength and negative psychological effects [9,10].

Body composition parameters, especially lean body mass, as well as patients’ nutritional status are considered to be important factors increasing the risk of major postoperative complications in cancer patients [11,12]. Aside from postoperative morbidity, impaired nutritional status is also shown to be associated with an increased length of hospital stay [13], decreased response to adjuvant treatment, such as chemotherapy and radiotherapy [14], and lower survival rates [15]. Notably, pancreatic cancer surgery is characterized by a high postoperative morbidity, reaching 40%, which significantly affects overall survival [16]. Under these circumstances, accurate predictive factors of postoperative morbidity and mortality could improve overall survival after pancreatic cancer surgery via tailored preoperative optimization. However, existing studies focus on isolated nutritional indices or single-center experiences, with few addressing a comprehensive range of preoperative nutritional and functional parameters in clinical settings.

The aim of this study is to evaluate the association between preoperative nutritional and functional status and short-term postoperative outcomes in patients undergoing surgery for pancreatic cancer. By incorporating both nutritional and functional markers in a two-center surgical cohort, this study aims to provide a more integrated risk assessment model that may inform targeted preoperative interventions and improve surgical outcomes.

## 2. Materials and Methods

### 2.1. Study Design

**Figure 1:**
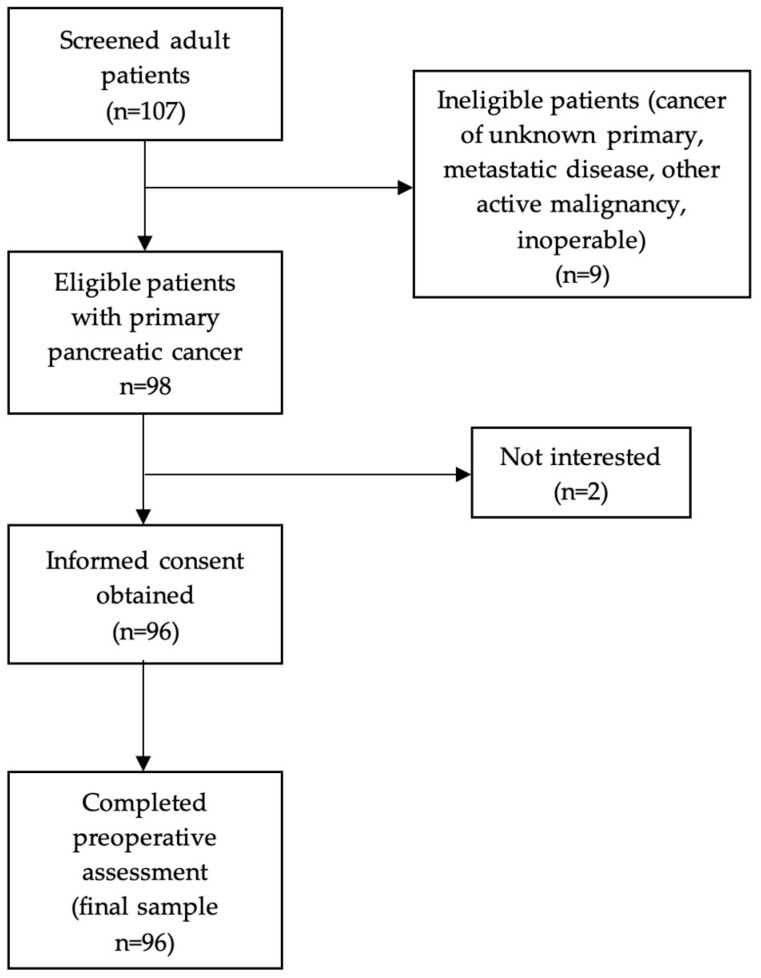
Flowchart of participants’ recruitment.

Eligible participants were adult individuals (aged ≥ 18 years) diagnosed with primary pancreatic cancer and admitted for surgery at two university-affiliated surgical departments in Athens, Greece (First Department of Surgery, Laikon General Hospital, and First Propaedeutic Department of Surgery, Hippocration General Hospital). Recruitment was conducted prospectively between September 2017 and October 2023. Inclusion in this study followed confirmation of the cancer diagnosis, and all participants provided written informed consent prior to enrollment. A comprehensive preoperative assessment, including nutritional and functional evaluation, was conducted by a trained clinical dietitian (Figure 1). The present study was conducted in compliance with the Declaration of Helsinki guidelines about ethical principles for medical research involving human subjects, and the study protocol was approved by the Human Research Ethics Committee of Laikon General Hospital (Ref. No. 696/11-11-2022) and Hippocration General Hospital (Ref. No. 3975/4-3-2022).

### 2.2. Patient Characteristics and Preoperative Assessment

Preoperative baseline data collection included demographic and clinical variables, such as age, sex, cancer type, and history of neoadjuvant therapy. Preoperative laboratory evaluation included analysis of fasting blood samples for hemoglobin (Hb), hematocrit (HCT), albumin, total lymphocyte count (TLC), neutrophil-to-lymphocyte ratio (NLR), and platelet-to-lymphocyte ratio (PLR). Information on the surgical procedure, adjuvant treatment, and cancer staging was documented after surgery. Staging was performed using the American Joint Committee on Cancer Staging Manual (8th edition) [17]. For the preoperative assessment of nutritional and functional status, weight and height were measured, and body mass index (BMI) was calculated as weight (kg) divided by the square of height (m^2^). Unintentional weight loss during the last 6 months was also recorded. Waist circumference (WC) was assessed by measuring waist diameter at a midpoint between the iliac crest and lower border of the tenth rib. Hip circumference (HC) was measured at the level of the widest circumference over the great trochanters. The average of two repeated measurements of WC and HC, respectively, was used in the analysis. Triceps skinfold (TSF) was measured directly over the triceps muscle on the posterior midline of the upper arm, at the midpoint between the acromial process of the scapula and the olecranon of the ulna using a caliper (Lange Skinfold Caliper).

The Patient-Generated Subjective Global Assessment (PG-SGA) was used to assess patients’ nutritional status prior to surgery. Patients were classified as (A) well nourished, (B) moderately malnourished/suspected malnutrition and (C) severely malnourished according to PG-SGA, as described in detail elsewhere [18,19].

The functional assessment included patients’ assessment of muscle strength and physical performance. Muscle strength was evaluated by handgrip strength of the dominant hand measured with a hand-held dynamometer (Takei 5401 Digital Dynamometer, Takei Scientific Instruments Co., Ltd., Niigata, Japan.). Three measurements were collected, and the mean value was used in all analyses [20]. In order to categorize patients into normal- or low-handgrip-strength groups, the cut-offs proposed by the European Working Group on Sarcopenia in Older People (EWGSOP) were used (i.e., handgrip strength < 27 kg for men and handgrip strength < 16 kg for women). Physical performance was assessed by measuring patient gait speed on a 4 m course; categorization into low and normal gait speed levels was based on EWGSOP criteria (cut-off speed ≤ 0.8 m/s) [21].

Furthermore, the Geriatric Nutritional Risk Index (GNRI) was also recorded preoperatively. GNRI [22] combines serum albumin levels with the ratio of actual body weight to ideal body weight. Lower GNRI scores are associated with higher degrees of malnutrition. Ideal body weight was calculated from the Lorentz formula (WLo). The GNRI formula is as follows: GNRI = [1.489 × albumin (g/L)] + [41.7 × (weight/WLo)]. The body weight/WLo weight is set to 1, when the patient’s body weight exceeds WLo. Patients were classified as a low GNRI group (GNRI < 92) and a high GNRI (GNRI ≥ 92) group. Prognostic Nutritional Index (PNI) was also calculated using the following formula: PNI = albumin (g/L) + 5 × absolute lymphocyte count (10^9^/L) [23].

### 2.3. Postoperative Outcomes Assessment

Surgical complications were categorized using the Clavien-Dindo classification Postoperative [24]. Complication categories I–II were classified as minor complications, whereas categories III–V were classified as major complications. Postoperative complications and morbidity were recorded during the initial hospital stay, from day of surgery to discharge. Complications resulting in patients being readmitted within 30 days of surgery were also assessed. Postoperative mortality was defined as any death occurring during hospitalization or within 90 days after surgery. Length of hospital stay (LOS) was defined as the time interval between surgery and discharge from hospital.

### 2.4. Statistical Analysis

Descriptive statistics were used to analyze frequencies, means, and standard deviations (SDs) of the study variables. Normality was assessed using the Shapiro–Wilk test. Subgroup comparisons of categorical variables were conducted using the chi-squared or Fisher’s exact test. Student’s *t*-test was used for comparing means of normally distributed continuous variables, whereas the Mann–Whitney U test was utilized for non-parametric comparisons. One-way ANOVA was applied for comparisons across multiple groups. Pearson’s correlation coefficient was used to examine associations between continuous variables. Multivariate analysis was conducted using binary logistic regression to identify independent predictors of major complications and mortality. Variables with a *p*-value ≤ 0.05 in the univariate analysis were entered into the model. Results are presented as odds ratios (OR) with 95% confidence intervals (CI). Model fit was assessed using Hosmer–Lemeshow test. Missing data were excluded from the analysis. Statistical significance was set at *p* ≤ 0.05. All analyses were performed using IBM SPSS Statistics for macOS, Version 28.0 (IBM Corp., Armonk, NY, USA).

## 3. Results

### 3.1. Baseline Characteristics of Study Participants

The baseline characteristics of study participants are summarized in Table 1. A total of 96 patients diagnosed with primary pancreatic cancer were included in this study. The mean age of the participants was 65.0 ± 12.3 years, with 59 individuals (61.5%) being male. Further, 66 patients (68.8%) underwent pancreaticoduodenectomy, 26 patients (27.1%) underwent distal pancreatectomy, 1 patient (1%) underwent wedge resection and 3 patients underwent non-specific surgical procedures. More specifically, one patient underwent distal pancreatectomy with left hemicolectomy due to invasion of the colon; one patient underwent a pancreaticoduodenectomy with distal gastrectomy due to gastric invasion; and one patient underwent total pancreatectomy with left adrenalectomy due to adrenal invasion. Twenty-four participants (27%) demonstrated low handgrip strength, while 24.1% exhibited reduced gait speed. Furthermore, based on the PG-SGA assessment, 31.5% were classified as moderately malnourished or at risk of malnutrition, while 35.9% were severely malnourished.

### 3.2. Postoperative Outcomes

The prevalence of postoperative complications is presented in Table 2. A total of 69 patients (71.9%) developed postoperative complications. Specifically, 42.7% of patients experienced minor complications, while 29.2% developed major complications. The mortality rate was 9.4%, while mean LOS was 17.5 ± 8.9 days.

### 3.3. Determinants of Overall, Major Postoperative Complications and Mortality

The determinants of overall and major postoperative complications, as well as mortality, are presented in Table 3. Patients who developed postoperative complications had significantly higher PG-SGA scores (9.6 ± 6.9 vs. 6.6 ± 4.9, *p* = 0.041) compared to those without complications. Moreover, complication rates were significantly higher among patients with indicators of poor nutritional or functional status: 87.9% of those classified as severely malnourished according to the PG-SGA developed complications compared to 62.7% of those without severe malnutrition (*p* = 0.015); 90.5% of patients with a low GNRI developed complications versus 66.2% of those with normal GNRI (*p* = 0.023); 91.7% of patients with low handgrip strength developed complications versus 61.5% of those with normal strength (*p* = 0.037); and 90.5% of patients with reduced gait speed experienced complications compared to 63.6% with normal gait speed (*p* = 0.027).

Statistically significant associations were also observed between the presence of major complications and advanced cancer stage (≥III vs. <III, 48% vs. 23.4%, *p* = 0.024), Hb (11.5 ± 1.9 vs. 12.8 ± 1.7 g/dL, *p* = 0.001), HCT (34.7 ± 5.4 vs. 38.4 ± 4.7%, *p* = 0.001), and severely malnourished patients (PG-SGA category C) vs. categories A and B (42.4% vs. 23.7%, *p* = 0.05). Moreover, patients with an advanced stage had a greater mortality rate (24.0% vs. 3.1%), as well as patients with lower Hb (10.6 ± 2.1 vs. 12.6 ± 1.7 g/dL, *p* = 0.002) and HCT (32.2 ± 6.0 vs. 37.9 ± 4.8%, *p* = 0.002). Higher PG-SGA scores (12.9 ± 6.7 vs. 8.3 ± 6.3, *p* = 0.045) and WC (102.7 ± 14.0 vs. 93.2 ± 11.3 cm, *p* = 0.049) were also significantly associated with an increased risk of postoperative mortality. Results from the multivariate analysis are presented in Supplementary Table S1 for major complications and Supplementary Table S2 for mortality.

### 3.4. Determinants of Length of Hospital Stay

Determinants of LOS are presented in Table 4. Patients who received neoadjuvant radiotherapy had longer LOS (31.5 ± 3.5 vs. 17.2 ± 8.7, *p* = 0.023). In addition, low handgrip strength (21.8 ± 10.7 vs. 16.5 ± 7.9 kg, *p* = 0.013) and gait speed (r = −0.223, *p* = 0.038) were associated with a longer length of hospital stay.

## 4. Discussion

In this study, we explored the association between nutritional and functional status and short-term postoperative outcomes in patients undergoing surgery for pancreatic cancer. Our findings indicate that patients who developed overall postoperative complications had significantly higher PG-SGA scores compared to those without complications. Additionally, complication rates were significantly higher among patients with indicators of poor nutritional or functional status, such as low GNRI, low handgrip strength and gait speed. Furthermore, severe malnutrition was associated with an increased risk of major complications, while a higher PG-SGA score was significantly linked to an increased risk of postoperative mortality. Reduced handgrip strength and slower gait speed were also associated with a longer hospital stay.

According to recent data [25], pancreatic cancer is one of the deadliest malignancies and ranks among the top three cancers, associated with severe nutritional impairment and high rates of cancer cachexia [26]. The timing of diagnosis is critical, as more than 50% of pancreatic cancers are detected at an advanced stage, when systemic adverse effects become difficult—or even impossible—to reverse [27]. Several early detection methods are currently available, including endoscopic techniques, genomic biomarkers, and proteomic biomarkers [28]. Both intrinsic and extrinsic factors contribute to the risk of pancreatic cancer. Intrinsic factors include hereditary conditions, cystic fibrosis, chronic pancreatitis, diabetes, pancreatic cysts, and hepatitis B and C infections, while extrinsic factors include cigarette smoking, obesity, physical inactivity, diet, coffee and alcohol consumption, and the use of certain medications [28].

In our study, the prevalence of preoperative severe malnutrition, as assessed by the PG-SGA questionnaire, was 35.9%, while 31.5% of patients were classified as moderately malnourished or at risk of malnutrition. Additionally, 27% of participants demonstrated low handgrip strength, and 24.1% exhibited reduced gait speed, consistent with findings from previous studies [29,30]. Unintentional weight loss over the last 6 months was reported by 5.9% of participants.

The preoperative nutritional status of cancer patients has gained significant attention due to its potential impact on postoperative outcomes, especially following major surgical procedures [31,32,33,34]. According to our results, 71.9% of study participants developed postoperative complications, with 42.7% experiencing minor complications and 29.2% developing major complications. The observed mortality rate was 9.4%. Notably, patients classified as severely malnourished based on the PG-SGA questionnaire had a significantly higher risk of developing overall (87.9% vs. 62.7%) and major postoperative complications (42.4% vs. 23.7%) compared to well-nourished or moderately malnourished/at-risk patients. In addition, higher PG-SGA scores were significantly associated with an increased risk of postoperative morbidity and mortality. In our study, the PG-SGA proved to be an effective tool for capturing patients’ postoperative outcomes, as it offers a comprehensive assessment of nutritional status. Unlike indices limited to objective measures such as weight loss or dietary intake, the PG-SGA also includes symptoms that hinder nutritional intake and evaluates functional capacity. This multidimensional approach enables the PG-SGA to more accurately identify the severity of malnutrition and its clinical implications, which directly impact postoperative recovery in this patient population. GNRI was also a significant predictor of postoperative outcomes in our cohort, as patients with low GNRI had higher rates of overall complications compared to those with normal GNRI (90.5% vs. 66.2%). Previous studies have also highlighted GNRI as a valuable nutritional index for cancer patients, with good predictive value for both short- and long-term outcomes [34,35,36].

Our analysis also demonstrated a significant association between patients’ anthropometric measurements and postoperative outcomes. Interestingly, WC was positively associated with an increased risk of postoperative mortality. Waist circumference is a well-established anthropometric measure widely used to assess visceral adiposity. While malnutrition and excessive weight loss are the primary concerns for pancreatic cancer patients’ nutritional health, excess abdominal fat may contribute to higher postoperative complication rates in gastrointestinal cancer patients [37,38]. A possible explanation is that central obesity increases intra-abdominal pressure, thereby elevating the risk of complications such as anastomotic leakage. Importantly, body mass index (BMI) was not significantly associated with these complications, suggesting that waist circumference may be a more reliable indicator of surgical risk. These findings highlight the importance of incorporating central obesity, as measured by waist circumference, into preoperative risk assessments.

Handgrip strength and gait speed are well-established tools for assessing functional status in cancer patients, offering valuable insight into muscle strength and physical performance. In our study, lower handgrip strength was associated with a longer length of hospital stay, while gait speed showed a weak negative correlation with LOS. Additionally, 91.7% of patients with low handgrip strength developed postoperative complications, compared to 61.5% of those with normal handgrip strength. Similarly, 90.5% of patients with reduced gait speed experienced complications, versus 63.6% of those with normal gait speed. These findings are consistent with a recent meta-analysis reporting that sarcopenic patients had a significantly longer hospital stay compared to non-sarcopenic patients (mean difference: 4.54 days; 95% CI: 2.49–6.59; *p* < 0.001; I^2^ = 93%) [39]. In line with this, two recent studies found that low handgrip strength [40] and reduced gait speed [41] were significantly associated with an increased risk of postoperative complications following pancreatic resection.

Regarding preoperative laboratory data, we identified total lymphocyte count (TLC), hemoglobin (Hb), and hematocrit (HCT) as significant prognostic factors for postoperative outcomes. Specifically, patients with lower Hb and HCT levels exhibited higher rates of major complications and increased mortality. Moreover, preoperative anemia has been associated with a higher 90-day comprehensive complication index following pancreatic resection [42,43]. Similarly, another study linked anemia with a composite outcome, including postoperative medical complications and an increased length of stay, after both open and laparoscopic colorectal resections [44]. However, it remains to be clarified whether anemia is an independent risk factor for poor outcomes or merely a marker of underlying chronic disease. Albumin, primarily a marker of inflammation, was found to be lower in patients with complications but was not a statistically significant prognostic marker for postoperative outcomes. Previous studies have yielded mixed results. Funakoshi et al. recently demonstrated that preoperative albumin levels were not associated with postoperative pancreatic fistula in patients undergoing distal pancreatectomy [45]. Conversely, another study found that preoperative albumin levels were significantly associated with increased postoperative complications when a threshold of 44.5 g/L was used [46]. Nonetheless, since albumin levels can be influenced by various factors, including hydration status, renal function, and liver function, these findings should be interpreted with caution.

Additionally, the cancer stage was significantly associated with both postoperative major complications and 90-day mortality. One possible explanation is that patients with advanced-stage cancer (stages III–IV) often undergo more complex surgical procedures for tumor resection, which may elevate the risk of adverse postoperative outcomes and, consequently, higher mortality rates. Furthermore, patients with advanced cancer frequently receive adjuvant therapies that can compromise immune function, increase susceptibility to infections, and delay postoperative recovery [47]. In our study, however, the type of surgical procedure was not a significant prognostic factor for postoperative outcomes, a finding that may be attributed to the relatively small sample size being divided into multiple categories of procedures, thereby reducing statistical power.

Although this study demonstrated the relationship between nutritional and functional status and postoperative outcomes, several limitations should be considered when interpreting the results. First, the cut-off values used to define reduced muscle strength and physical performance were based on predefined thresholds that may not be fully tailored to our specific study population. Additionally, potential confounding variables, such as preoperative comorbidities and variations in surgical techniques, were not extensively analyzed. Moreover, this study focused on short-term clinical outcomes—specifically, the rate of postoperative complications and length of hospital stay—while long-term outcomes, including survival in relation to nutritional status, were not assessed. Furthermore, although the majority of patients had pancreatic ductal adenocarcinoma, a small number of individuals with other pancreatic cancer subtypes were included, which may have introduced heterogeneity. Another limitation is the lack of data on nutritional and functional status prior to neoadjuvant therapy, which could significantly affect patients’ nutritional and functional status. Finally, the limited sample size restricts the generalizability of our findings. Future large-scale, multi-institutional studies are needed to validate and expand upon these results.

## 5. Conclusions

Integrating patients’ nutritional and functional status into preoperative risk assessment may improve the prediction of unfavorable postoperative outcomes in those undergoing pancreatic surgery, as malnutrition is closely associated with poorer recovery and higher complication rates. Prehabilitation programs that incorporate individualized nutritional support and targeted exercise regimens can potentially enhance postoperative recovery. Future research should prioritize the development of such targeted prehabilitation strategies to optimize nutritional status before surgery, ultimately improving postoperative outcomes and long-term prognosis.

## Figures and Tables

**Table 1:** Baseline characteristics of study participants.

Characteristic	Patients (n = 96)
Age (years)	65.0 ± 12.3 65.0 ± 12.3 (Median: 67, Min: 27, Max: 89)
Sex	n (%)
Male	59 (61.5)
Female	37 (38.5)
Type of cancer (n = 89)	n (%)
Adenocarcinoma	83 (93.3)
NET	4 (4.5)
Mixed adenoneuroendocrine carcinoma	2 (2.2)
Surgical procedure	n (%)
Pancreaticoduodenectomy	66 (68.8)
Distal pancreatectomy	26 (27.1)
Wedge resection	1 (1.0)
Non specific	3 (3.1%)
Neoadjuvant Chemotherapy	n (%)
Yes	20 (20.8)
No	76 (79.2)
Neoadjuvant Radiotherapy	n (%)
Yes	2 (2.1)
No	94 (97.9)
Stage (n = 89)	n (%)
in situ	5 (5.6)
I	17 (19.1)
II	44 (49.4)
III	19 (21.3)
IV	4 (4.5)
Adjuvant Chemotherapy (n = 76)	n (%)
Yes	47 (61.8)
No	29 (38.2)
Adjuvant Radiotherapy (n = 76)	n (%)
Yes	3 (3.9)
No	73 (96.1)
Albumin (g/dL)	3.8 ± 0.8
Hb (g/dL)	12.4 ± 1.9
HCT (%)	37.4 ± 5.2
TLC (K/μL)	2.0 ± 1.8
NLR (n = 94)	3.35 ± 4.08
PLR (n = 95)	170.8 ± 145.4
PG-SGA (n = 92)	8.8 ± 6.5
PG-SGA category (n = 92)	n (%)
A	30 (32.6)
B	29 (31.5)
C	33 (35.9)
GNRI (n = 95)	103.7 ± 17.9
PNI (n = 95)	38.8 ± 7.0
BMI (kg/m^2^)	25.8 ± 3.8
WC (cm)	93.9 ± 11.6
HC (cm)	98.0 ± 10.1
WHR	0.96 ± 0.07
TSF (mm)	10.3 ± 11.7
Weight Loss (%)	5.9 ± 5.9
Handgrip strength (kg)	27.1 ± 10.6
Handgrip strength category (n = 89)	n (%)
Low	24 (27.0)
Normal	65 (73.0)
Gait speed (m/s) (n = 58)	0.97 ± 0.32
Gait speed category (n = 87)	n (%)
Low	21 (24.1)
Normal	66 (75.9)

NET: neuroendocrine tumor; Hb: hemoglobin, HCT: hematocrit; TLC: total lymphocyte count; NLR: neutrophil-to-lymphocyte ratio; PLR: platelet-to-lymphocyte ratio; PG-SGA: Patient-Generated Subjective Global Assessment; GNRI: Geriatric Nutritional Risk Index; PNI: Prognostic Nutritional Index; BMI: Body mass index; WC: waist circumference; HC: hip circumference; WHR: waist-to-hip ratio; TSF: triceps skinfold.

**Table 2:** Prevalence of postoperative complications.

Postoperative Complications	Patients (n = 96)
Complication	n (%)
Yes	69 (71.9)
No	27 (28.1)
Clavien grade	n (%)
0	27 (28.1)
I	6 (6.3)
II	35 (36.5)
III	17 (17.7)
IV	2 (2.1)
V	9 (9.4)
Minor complications (Ι–ΙΙ)	41 (42.7)
Major complications (ΙΙΙ–V)	28 (29.2)
Mortality	
Yes	9 (9.4%)
No	87 (90.6%)
LOS (days)	17.5 ± 8.9

**Table 3:** Determinants of overall, major postoperative complications and mortality.

Parameter	Complications			Major Complications			Mortality		
Variable	Yes (n = 69)	No (n = 27)	*p*-value	Yes (n = 28)	No (n = 68)	*p*-value	Yes (n = 9)	No (n = 87)	*p*-value
Age (years)	65.5 ± 12.8	63.8 ± 2.2	0.553	67.29 ± 13.6	64.0 ± 11.7	0.241	72.2 ± 9.5	64.2 ± 12.3	0.063
Sex	n (%)	n (%)	0.303	n (%)	n (%)	0.449	n (%)	n (%)	1.000
Male	44 (74.6)	15 (25.4)		18 (30.5)	41 (69.5)		6 (10.2)	53 (89.8)	
Female	25 (67.6)	12 (32.4)		10 (27.0)	27 (73.0)		3 (8.1)	34 (91.9)	
Type of cancer (n = 89)	n (%)	n (%)	0.072	n (%)	n (%)	0.324	n (%)	n (%)	0.382
Adenocarcinoma	65 (78.3)	18 (21.7)		25 (30.1)	58 (69.9)		8 (9.6)	75 (90.4)	
NET	2 (50.0)	2 (50.0)		1 (25.0)	3 (75.0)		0 (0)	4 (100.0)	
Mixed adenoneuroendocrine carcinoma	2 (100)	0 (0)		2 (100)	0 (0)		1 (50.0)	1 (50.0)	
Surgical procedure	n (%)	n (%)	0.595	n (%)	n (%)	0.512	n (%)	n (%)	0.943
Pancreaticoduodenectomy	45 (68.2)	21 (31.8)		19 (28.8)	47 (71.2)		7 (10.6)	59 (89.4)	
Distal Pancreatectomy	21 (80.8)	5 (19.2)		7 (26.9)	19 (73.1)		2 (7.7)	24 (92.3)	
Wedge resection	1 (100.0)	0 (0)		0 (0)	1 (100)		0 (0)	1 (100)	
Non specific	2 (66.7)	1 (33.3)		2 (66.7)	1 (33.3)		0 (0)	3 (100)	
Neoadjuvant Chemotherapy	n (%)	n (%)	0.412	n (%)	n (%)	0.375	n (%)	n (%)	0.291
Yes	16 (80.0)	4 (20.0)		7 (35.0)	13 (65.0)		3 (15.0)	17 (85.0)	
No	53 (69.7)	23 (30.3)		21 (27.6)	55 (72.4)		6 (7.9)	70 (92.1)	
Neoadjuvant Radiotherapy	n (%)	n (%)	0.486	n (%)	n (%)	0.500	n (%)	n (%)	1.000
Yes	1 (50.0)	1 (50.0)		1 (50.0)	1 (50.0)		0 (0)	2 (100)	
No	68 (72.3)	26 (27.7)		27 (28.7)	67 (71.3)		9 (9.6)	85 (90.4)	
Stage (n = 89)	n (%)	n (%)	0.433	n (%)	n (%)	0.024	n (%)	n (%)	0.006
≤II	45 (70.3)	19 (29.7)		15 (23.4)	49 (76.6)		2 (3.1)	62 (96.9)	
III–IV	20 (80.0)	5 (20.0)		12 (48.0)	13 (52.0)		6 (24.0)	19 (76.0)	
Hb (g/dL)	12.2 ± 1.9	13.01 ± 1.6	0.052	11.5 ± 1.9	12.8 ± 1.7	0.001	10.6 ± 2.1	12.6 ± 1.7	0.002
HCT (%)	36.7 ± 5.4	39.0 ± 4.3	0.055	34.7 ± 5.4	38.4 ± 4.7	0.001	32.2 ± 6.0	37.9 ± 4.8	0.002
Albumin (g/dL)	3.7 ± 0.9	4.1 ± 0.5	0.058	3.7 ± 0.8	3.9 ± 0.8	0.348	3.4 ± 1.1	3.9 ± 0.8	0.066
TLC (K/μL)	1.8 ± 0.8	2.4 ± 3.2	0.142	1.7 ± 0.9	2.1 ± 2.0	0.260	2.3 ± 1.2	2.0 ± 1.9	0.608
NLR	3.26 ± 2.35	3.59 ± 6.7	0.724	3.78 ± 4.59	3.17 ± 4.59	0.516	4.08 ± 3.13	3.28 ± 4.18	0.575
PLR	155.52 ± 71.16	209.15 ± 47.64	0.105	178.95 ± 77.43	167.34 ± 166.23	0.725	130.77 ± 71.95	174.95 ± 150.72	0.389
PG-SGA (n = 92)	9.6 ± 6.9	6.6 ± 4.9	0.041	10.4 ± 7.1	8.0 ± 6.1	0.106	12.9 ± 6.7	8.3 ± 6.3	0.045
PG-SGA category (n = 92)	n (%)	n (%)	0.015	n (%)	n (%)	0.05	n (%)	n (%)	0.065
A + B	37 (62.7)	22 (37.3)		14 (23.7)	45 (76.3)		3 (5.1)	56 (94.9)	
C	29 (87.9)	4 (12.1)		14 (42.4)	19 (57.6)		6 (18.2)	27 (81.8)	
GNRI (n = 95)	102.7 ± 19.0	106.3 ± 14.8	0.382	104.9 ± 15.8	103.2 ± 18.8	0.674	98.3 ± 20.1	104.3 ± 17.7	0.340
GNRI category (n = 95)	n (%)	n (%)	0.023	n (%)	n (%)	0.425	n (%)	n (%)	0.105
high (≥92)	49 (66.2)	25 (33.8)		21 (28.4)	53 (71.6)		5 (6.8)	69 (93.2)	
low (<92)	19 (90.5)	2 (9.5)		7 (33.3)	14 (66.7)		4 (19.0)	17 (81.0)	
PNI	38.0 ± 7.4	40.9 ± 5.3	0.069	37.2 ± 8.0	39.5 ± 6.5	0.149	33.7 ± 10.2	39.3 ± 6.4	0.141
BMI (kg/m^2^)	26.1 ± 3.9	25.2 ± 3.8	0.357	26.5 ± 3.7	25.5 ± 3.9	0.276	25.7 ± 4.2	25.8 ± 3.9	0.941
WC (cm)	94.7 ± 11.4	96.7 ± 11.5	0.349	97.4 ± 11.3	92.5 ± 11.6	0.079	102.7 ± 14.0	93.2 ± 11.3	0.049
HC (cm)	98.6 ± 9.4	96.7 ± 11.5	0.423	99.9 ± 9.7	97.3 ± 10.2	0.290	96.4 ± 13.4	98.1 ± 10.0	0.712
TSF (mm)	11.1 ± 12.5	8.5 ± 9.6	0.342	13.0 ± 13.5	9.2 ± 10.8	0.160	12.3 ± 11.4	10.1 ± 11.8	0.617
Weight Loss (%)	6.5 ± 5.4	4.5 ± 7.0	0.153	6.9 ± 5.1	5.4 ± 6.2	0.282	8.8 ± 3.7	5.6 ± 6.0	0.128
Handgrip strength (kg)	26.3 ± 10.8	28.9 ± 10.2	0.284	25.6 ± 12.2	27.6 ± 10.0	0.418	21.4 ± 15.6	27.5 ± 10.1	0.144
Handgrip strength category (n = 89)	n (%)	n (%)	0.037	n (%)	n (%)	0.097	n (%)	n (%)	0.082
Low	22 (91.7)	2 (8.3)		10(41.7)	14 (58.3)		4 (16.7)	20 (83.3)	
Normal	40 (61.5)	25 (38.4)		16(24.6)	49 (75.4)		3 (4.6)	62 (95.4)	
Gait speed (m/s)	0.91 ± 0.34	1.10 ± 0.23	0.004	0.92 ± 0.36	0.99 ± 0.30	0.329	1.02 ± 0.48	0.97 ± 0.31	0.698
Gait speed category (n = 87)	n (%)	n (%)	0.027	n (%)	n (%)	0.088	n (%)	n (%)	0.628
Low	19 (90.5)	2 (9.5)		9 (42.9)	12 (57.1)		2 (9.5)	19 (90.5)	
Normal	42 (63.6)	24 (36.4)		16 (24.2)	50 (75.8)		4 (6.1)	62 (93.9)	

NET: neuroendocrine tumor; Hb: hemoglobin, HCT: hematocrit; TLC: total lymphocyte count; NLR: neutrophil-to-lymphocyte ratio; PLR: platelet-to-lymphocyte ratio; PG-SGA: Patient-Generated Subjective Global Assessment; GNRI: Geriatric Nutritional Risk Index; PNI: Prognostic Nutritional Index; BMI: Body mass index; WC: waist circumference; HC: hip circumference; WHR: waist-to-hip ratio; TSF: triceps skinfold.

**Table 4:** Determinants of length of hospital stay.

Parameter	LOS	*p*-Value
Age (years)	0.061	0.557
Sex		0.101
Male	19.3 ±10.8	
Female	16.3 ± 7.2	
Type of cancer (n = 89)		0.254
Adenocarcinoma	16.7 ± 8.8	
NET	17.8 ± 8.7	
Mixed adenoneuroendocrine carcinoma	17.9 ± 9.2	
Surgical procedure		0.717
Pancreaticoduodenectomy	16.4 ± 7.4	
Distal Pancreatectomy	13.0 ± 0.0	
Wedge resection	21.7 ±13.6	
Non specific	17.7 ± 9.3	
Neoadjuvant Chemotherapy		0.688
Yes	18.3 ± 9.5	
No	17.4 ± 8.8	
Neoadjuvant Radiotherapy		0.023
Yes	31.5 ±3.5	
No	17.2 ± 8.7	
Stage (n = 89)		0.695
≤II	17.8 ± 8.3	
III–IV	17.0 ± 10.6	
Hb (g/dL)	−0.142	0.169
HCT (%)	−0.175	0.089
Albumin (g/dL)	−0.031	0.767
TLC (K/μL)	−0.070	0.502
NLR	−0.066	0.529
PLR	−0.004	0.970
PG-SGA (n = 92)	0.039	0.709
PG-SGA category (n = 92)		0.580
A + B	18.0 ± 9.2	
C	16.9 ± 8.8	
GNRI (n = 95)	0.019	0.858
GNRI category (n = 95)		0.979
high (≥92)	17.5 ± 8.8	
low (<92)	17.6 ± 9.2	
PNI	−0.065	0.532
BMI (kg/m^2^)	0.003	0.975
WC (cm)	0.132	0.229
HC (cm)	0.175	0.111
TSF (mm)	0.069	0.514
Weight Loss (%)	−0.157	0.270
Handgrip strength (kg)	−0.210	0.049
Handgrip strength category (n = 89)		0.013
Low	21.8 ± 10.7	
Normal	16.5 ± 7.9	
Gait speed (m/s)	−0.223	0.038
Gait speed category (n = 87)		0.151
Low	20.2 ± 10.1	
Normal	16.9 ± 8.6	

NET: neuroendocrine tumor; Hb: hemoglobin, HCT: hematocrit; TLC: total lymphocyte count; NLR: neutrophil-to-lymphocyte ratio; PLR: platelet-to-lymphocyte ratio; PG-SGA: Patient-Generated Subjective Global Assessment; GNRI: Geriatric Nutritional Risk Index; PNI: Prognostic Nutritional Index; BMI: Body mass index; WC: waist circumference; HC: hip circumference; WHR: waist-to-hip ratio; TSF: triceps skinfold.

## Data Availability

Data are available from the corresponding author.

## Supplementary Materials

The following supporting information can be downloaded at: https://www.mdpi.com/article/10.3390/life15050803/s1, Table S1: Multivariate Logistic Regression Analysis for Major Postoperative Complications; Table S2. Multivariate Logistic Regression Analysis for Postoperative Mortality.
