# Supplementary material for: Exploring the Link Between Nutritional and Functional Status and Short-Term Postoperative Outcomes in Patients Undergoing Pancreatic Cancer Surgery
Source: Life (Basel). 2025 May 18;15(5):803. doi: 10.3390/life15050803 (PMC12112883; doi:10.3390/life15050803)
Supplement: Supplementary file 1 [file life-15-00803-s001.zip › life-3633272-supplementary.pdf]

Supplementary Table S1. Multivariate Logistic Regression Analysis for Major Postoperative Complications

Variable	B	S.E.	Wald	df	p-value	OR (Exp(B))	95% CI
Stage (I–II vs III–IV)	0.814	0.553	2.170	1	0.141	2.258	0.764 – 6.672
Hemoglobin (Hb)	-0.331	0.160	4.275	1	0.039	0.718	0.525 – 0.983
PG-SGA Category (1 vs 2–3)	0.329	0.538	0.373	1	0.542	1.389	0.484 – 3.990
Constant	2.898	2.066	1.967	1	0.161	18.134	–

OR: odds ratio

Supplementary Table S2. Multivariate Logistic Regression Analysis for Postoperative Mortality

Variable	B	S.E.	Wald	df	p-value	OR (Exp(B))	95% CI for OR
Stage (I–II vs III–IV)	1.426	1.003	2.022	1	0.155	4.162	0.583 – 29.709
Hemoglobin (Hb)	-0.259	0.354	0.535	1	0.465	0.772	0.386 – 1.545
Waist Circumference (WC)	0.059	0.048	1.527	1	0.217	1.061	0.966 – 1.166
PG-SGA Score	0.053	0.080	0.426	1	0.514	1.054	0.900 – 1.234
Constant	-6.451	6.946	0.863	1	0.353	0.002	–

OR: odds ratio
